# Immortalization of patient-derived lip cells for establishing 3D lip models

**DOI:** 10.3389/fcell.2024.1449224

**Published:** 2024-11-04

**Authors:** Farah Mansour, Ludovica Parisi, Silvia Rihs, Isabelle Schnyder, Giorgio C. La Scala, Nijas Aliu, Christos Katsaros, Martin Degen

**Affiliations:** ^1^ Laboratory for Oral Molecular Biology, Department of Orthodontics and Dentofacial Orthopedics, University of Bern, Bern, Switzerland; ^2^ Graduate School for Cellular and Biomedical Sciences, University of Bern, Bern, Switzerland; ^3^ University Clinic for Pediatric Surgery, Bern University Hospital, Bern, Switzerland; ^4^ Division of Pediatric Surgery, Department of Pediatrics, University Hospital of Geneva, Geneva, Switzerland; ^5^ Department of Human Genetics, Bern University Hospital, Inselspital, University of Bern, Bern, Switzerland

**Keywords:** cleft lip, lip, keratinocyte, cell line, cell differentiation, 3D-modeling

## Abstract

**Introduction:**

The lips fulfill various critical physiological roles besides being viewed as a fundamental aesthetic feature contributing to the perception of health and beauty. Therefore, any lip injury, abnormality, or congenital malformation, such as cleft lip, needs special attention in order to restore proper lip function and aesthetics. To achieve this goal, a better understanding of the complex lip anatomy, function, and biology is required, which can only be provided by basic research endeavors. However, the current lack of clinically relevant human lip cells and three-dimensional *in vitro* lip models, capable of replacing ethically questionable animal experimentations, represents a significant limitation in this area of research.

**Methods:**

To address these limitations, we aimed to pioneer the introduction of immortalized healthy lip- and cleft lip-derived keratinocytes. Primary keratinocytes were isolated from patients’ samples and immortalized by introducing the catalytic domain of telomerase, combined with the targeted knockdown of the cell cycle inhibitor gene, *p16* ^*INK4A*^. We then focused on validating the newly established cell lines by comparing their genetic stability and key phenotypic features with their primary keratinocyte counterparts.

**Results:**

The newly established immortalized keratinocyte cell lines demonstrated genetic stability and preserved the main phenotypic characteristics of primary keratinocytes, such as cellular morphology and differentiation capacity. Three-dimensional lip models, generated using these cell lines, proved to be effective and convenient platforms for screening applications, including wound healing and microbial infection of the lip epithelium.

**Discussion:**

The establishment of immortalized keratinocytes derived from healthy and cleft lips represents a significant achievement in lip research. These cell lines and the associated three-dimensional lip models are valuable tools that can be used as convenient screening platforms for various assays in a multitude of lip-related research areas, including dermatology, skin care, wound healing, tissue engineering, and craniofacial anomalies. This work opens new avenues in studying lip abnormalities and provides unique tools for personalized medicine approaches beneficial to patients.

## 1 Introduction

The lips, comprising a delicate transition between the facial skin and the oral cavity, are prominent features of the human face, serving essential roles in speech articulation, facial expression, and sensory perception. Beyond their functional attributes, lips also hold aesthetic significance, contributing to personal appearance. Accordingly, lip abnormalities resulting from congenital malformations, traumatic lesions, age-related changes, manifestations of infections and inflammatory diseases, or tumors, are readily noticeable and frequently associated with psychological stress (Greenberg et al., 2017).

Orofacial clefts (OFC) are the most frequent congenital anomalies involving the lips, and can be either limited to the upper lip or extend into the palate, manifesting in cleft lip with or without cleft palate (CLP). Cleft lip-affected patients require a surgical repair at the age of 3–6 months, followed by a long-term multidisciplinary therapy regimen. Still, the final aesthetic and functional outcomes are not always predictable and satisfying, contributing to the significant psychosocial, emotional, and economic burden that OFC patients and their families experience (Wehby and Cassell, 2010).

To advance the discovery of novel and personalized treatment options concerning lip abnormalities, clinically relevant *in vitro* cell models are indispensable. Primary cells are optimal for studying biological functions as they are believed to retain original tissue characteristics (Pan et al., 2009). However, they display strict limitations in their ability to proliferate *ex vivo*, and consequently, they are highly restricted in their availability. This, combined with additional drawbacks makes research with primary cells challenging, especially if studying lip tissue, which is not routinely biopsied and for which only small tissue remnants can be obtained.

The irreversible growth arrest of primary cells in culture is known as cellular senescence, which *in vitro* can be classified as replicative and stress-induced senescence (Sedivy, 1998; Ramirez et al., 2001). Replicative senescence is characterized by the progressive shortening of telomeres after each cell division, eventually leading to telomeres length falling below a critical threshold, the Hayflick Limit (Shay and Wright, 2000). In contrast, stress-induced senescence is associated with the upregulation of the tumor suppressor and cell cycle inhibitor p16^INK4A^/Retinoblastoma (Rb) pathway and is induced by exogenous cellular insults (*e.g*., suboptimal culture conditions). *Ex vivo*, the limited lifespan of primary cells can be bypassed by either the overexpression of oncogenes such as Ras (Kelekar and Cole, 1987), the expression of specific viral proteins [*e.g*., papillomavirus E6 and E7 proteins (Munger et al., 1989)], or the ectopic introduction of the catalytic subunit of telomerase (hTERT), which counteracts the progressive loss of telomeres after each DNA replication cycle (Counter et al., 1998). However, in keratinocytes, expression of hTERT alone is insufficient to enable evasion from senescence. To achieve their immortalization, disruption of the p16^INK4A^/Rb-pathway in combination with hTERT expression is required (Dickson et al., 2000).

Since patient-derived cells are the frontrunners for developing clinically relevant and personalized human *in vitro* models, we are routinely isolating and biobanking such cells from regularly discarded tissues. Notably, our living cell repository consists of primary keratinocytes and their corresponding fibroblasts isolated from a multitude of tissue biopsies mainly originating from the cranio-/orofacial region and predominantly comprises cells sourced from tissue remnants obtained during cleft lip surgery (Parisi et al., 2021). However, since the lip biopsies are of limited size, only a finite number of cells can be archived and used for experiments. To avoid the depletion of valuable and unique clinical specimens, and to simplify the practicability of working with primary cells, we sought to immortalize lip-derived keratinocytes from both a healthy and a non-syndromic CLP donor as an initial proof-of-concept study. Since retention of the original properties within the immortalized cells represents a strict prerequisite for clinical relevance, a thorough comparison between intraindividual native tissue, primary, and immortalized keratinocytes is imperative. These analyses revealed that the newly established lip keratinocyte cell lines were indistinguishable from their corresponding parental primary cells regarding typical keratinocyte traits. The availability of lip-derived keratinocytes that display an extended life span are easy to handle, and retain the original tissue properties, opens new intriguing avenues for modeling and studying lip-associated defects. This might represent an important step toward the development of optimized and personalized treatment options and/or potentially preventive strategies for various lip abnormalities, including but not limited to OFCs.

## 2 Materials and methods

### 2.1 Ethics statement

This work was performed according to the Ethical Principles for Medical Research Involving Human Subjects as defined by the World Medical Association (WMA Declaration of Helsinki - Ethical principles for medical research involving human subjects). Isolation of human cleft lip-as well as control tissue-derived cells and their analyses for this study have been approved by the Kantonale Ethikkommission of Bern, Switzerland (protocol number: 2017–01394). Written informed consent was obtained from the legal representatives of the children.

### 2.2 Cell culture

Primary keratinocytes were isolated from lip biopsies using the explant culture technique (Degen et al., 2018; Parisi et al., 2021). Cleft lip-derived keratinocytes (Pa-Ep) were gained from discarded tissue along the cleft margins during cheiloplasty, while healthy lip keratinocytes (19K-Ep) were obtained from a healthy donor sustaining a lip laceration (Degen et al., 2020). All keratinocytes (primary and immortalized) were cultured in Keratinocyte Serum-Free Medium (KSFM, Gibco, Thermo Fisher Scientific, Waltham, MA, United States) supplemented with 25 μg/mL bovine pituitary extract (BPE), 0.2 ng/mL epidermal growth factor (EGF), 0.3 mM CaCl_2_ (unless specified otherwise), and 1×Penicillin/Streptomycin (P/S, GIBCO, Thermo Fisher Scientific) as described elsewhere (Degen et al., 2013). Phoenix retroviral packaging cells and the breast cancer cell line T47D (ATCC#HTB-133™) were cultured in Dulbecco’s Modified Eagle Medium (DMEM, high glucose, Thermo Fisher Scientific) supplemented with 10% fetal bovine serum and 1×P/S. Details of the cells used in this study are reported in Supplementary Table S1.

### 2.3 Retroviral vectors and transduction

The retroviral vector plasmid MSCV-pic2neo-hTERT-p16 shRNA, a gift from Ji Luo (Addgene plasmid #164920), was transfected into Phoenix cells using Viafect (Promega, Dübendorf, Switzerland). After 18 h, the transfection mix was replaced with a 1:1 (vol/vol) mixture of KSFM and “DF-K medium”, the latter consisting of a 1:1 mixture of Ca^2+^-free, glutamine-free DMEM and Ham’s F-12 (Thermo Fisher Scientific) and supplemented with 0.2 ng/mL EGF, 25 μg/mL BPE, 1.5 mM L-glutamine, and 1×P/S (Degen et al., 2012). Virus-containing supernatants were harvested 40 and 48 h post-transfection, passed through a 0.45 μm pore filter, and stored at −80°C until use. Keratinocytes plated 1 day previously at 1.2 × 10^5^ cells/9.5 cm^2^ well in KSFM were transduced for 6 h with retroviral supernatant in the presence of 2 μg/mL polybrene (Sigma-Aldrich, St. Louis, MO, United States). Transduced keratinocytes were sub-cultured the next day in KSFM containing 0.2 mg/mL G418 (Sigma-Aldrich) and drug-selected for 8 days to obtain stable transductants.

### 2.4 RNA, cDNA, and quantitative real-time polymerase chain reaction (qPCR)

Total RNA was purified using the innuPREP RNA Mini kit (IST Innuscreen GmbH, Berlin, Germany) according to the manufacturer’s protocol for eukaryotic cells. Five hundred ng of RNA were used as template for synthesizing the first strand cDNA using an Oligo (dT)_15_ primer and the M-MLV Reverse Transcriptase (Promega). Gene expression was analyzed by qPCR using the GoTaq^®^ qPCR Master Mix (Promega) on a QuantStudio 3 instrument (Applied Biosystems, Thermo Fisher Scientific). Values were normalized to Glyceraldehyde 3-phosphate dehydrogenase (*GAPDH)* by applying either the ΔCt method for absolute levels or the ΔΔCt for levels further normalized to a control sample set to 1. Primers used for qPCR primers were designed using the NCBI primer tool (www.ncbi.nlm.nih.gov/tools/primer-blast/) and are shown in Supplementary Table S2.

### 2.5 Immunoblotting

Keratinocytes were lysed in 1×RIPA buffer (10 mM Tris-HCl (pH 8.0), 1 mM EDTA, 0.1% sodium deoxycholate, 0.1% SDS, 1% NP40, 140 mM NaCl) supplemented with cOmplete™ Mini Protease Inhibitor Cocktail and PhosSTOP™ EASYpack (both from Sigma-Aldrich). Total protein concentration was determined using the BCA Protein Assay Kit (Pierce, Thermo Fisher Scientific). Fifteen μg of protein lysate in loading buffer (62.6 mM Tris-HCl (pH 6.8), 2% SDS, 10% glycerol, 0.01% bromophenol blue) containing 100 mM dithiothreitol were boiled for 5 min at 95°C, separated by Sodium dodecyl-sulfate polyacrylamide gel electrophoresis under reducing conditions, blotted on to polyvinylidene difluoride membranes, and blocked for 1 h at room temperature (RT) in Tris-buffered saline/0.05% Tween-20 (TBS-T) and 5% skim milk powder. Afterward, membranes were incubated with the primary antibodies overnight at 4°C, washed in TBS-T, and incubated with horseradish peroxidase (HRP)-conjugated anti-rabbit or -mouse IgG for 1 h at RT. Finally, blots were developed using SuperSignal West Dura or Pico substrate (Pierce, Thermo Fisher Scientific) and scanned on a Chemi Premium imager (VWR, Darmstadt, Germany). Some of the immunoblots were analyzed densitometrically using the ImageJ software (https://imagej.net/ij/). Briefly, the intensity of each protein band was normalized to the vinculin internal control of the same extract in the same experiment. Antibodies used are listed in Supplementary Table S3.

### 2.6 Lifespan

To measure the replicative lifespan, keratinocytes were plated at a density of 10^4^ cells/21.5 cm^2^ in KSFM and cultured for 5–7 days with regular re-feedings. Population doublings (PDs) were calculated as log_2_ (# cells at the time of sub-culture/# cells plated), and cumulative PDs were determined by summing the individual PDs for each passage. Re-seeding of original tissue outgrowths was labeled as passage 1.

### 2.7 Immunofluorescence (IF)

Keratinocyte cultures were fixed in 4% buffered formaldehyde (Grogg Chemie, Stettlen, Switzerland) for 20 min at RT, before being washed in phosphate-buffered saline (PBS) and permeabilized in 0.1% Triton-X-100 (Sigma-Aldrich) for 5 min. After blocking the samples for 30 min in 3% Bovine Serum Albumin (BSA)/TBS-T, samples were incubated for 2 h at RT with primary antibodies (Supplementary Table S3), rinsed thoroughly with PBS, followed by incubation with fluorescent-labeled secondary antibodies (Molecular Probes, Thermo Fisher Scientific) with or without tetramethylrhodamine (TRITC)-phalloidin (Tocris, Bio-Techne, Minneapolis, MN, United States) for 1 h, light-protected. Finally, cells were washed three times with PBS and once with double-distilled (dd) H_2_O before being coverslip-mounted with Vectashield Mounting Medium containing DAPI (Vector Laboratories, Burlingame, CA, United States). Samples were examined under an Olympus BX51 phase/fluorescence microscope (Olympus Life Science Solutions, Tokyo, Japan) equipped with a xenon lamp (X-Cite, series 120 PC Q, Lumen Dynamics, Mississauga, Canada) and fluorescence filters U-MWIBA3 for AlexaFluor 488, U-MWIGA3 for TRITC, and U-MNUA2 for DAPI (Olympus Life Science Solutions). Images were acquired with a ProgRes C5 camera with ProgRes CapturePro software (Jenaoptik, Jena, Germany). ImageJ was used to quantify p16^INK4A^ fluorescence intensity by measuring the mean gray value, with background noise subtracted. Single-cell areas and colony circularities (cc) were quantified using ImageJ. cc = 4π(A/P^2^) (A: colony area, P: colony perimeter).

### 2.8 Measurement of telomerase activity

Telomerase activity was analyzed by the Telomerase Repeated Amplification protocol (TRAP) as described by Mender and Shay (Mender and Shay, 2015). In brief, 10^5^ cells were lysed in 40 μL NP40 lysis buffer and stored at −80°C until use. One μl of cell lysate was added to the fragment amplification reaction using a primer mix including the primers ACX (5′-GCG CGG CTT ACC CTT ACC CTT ACC CTA ACC-3′), NT (5′- ATC GCT TCT CGG CCT TTT-3′), TSNT (5′-AAT CCG TCG AGC AGA GTT AAA AGG CCG AGA AGC GAT-3′) (Microsynth, Balgach, Switzerland), and telomeres were extended by incubating the reaction at 25°C for 40 min followed by 2 min at 95°C to deactivate the telomerase. Thereafter, PCR was run for 25 cycles (95°C for 30 s, 52°C for 30 s, 72°C for 45 s) with a final elongation step at 72°C for 10 min. PCR products were separated on a 10% acrylamide (Fisher Scientific) (19:1 acrylamide:bis-acrylamide) in TRIS-Borat-EDTA buffer. Gels were stained with SYBR safe DNA Gel Stain (Thermo Fisher Scientific) and visualized under UV light.

### 2.9 Hypermotility and growth inhibition assays

Growth inhibition and hypermotility assays were performed as previously described (Natarajan et al., 2006). Briefly, keratinocytes were seeded at a density of 1,000 cells/9.5 cm^2^ in either regular KSFM containing 0.03 ng/mL transforming growth factor β1 (“TGFβ1”, Proteintech, Manchester, United Kingdom), in KSFM supplemented only with 0.3 mM Ca^2+^ and 12.5 μg/mL BPE (”-EGF”), or on dishes precoated with fibroblast conditioned medium (“ECM-coated”) in regular KSFM. Coating was performed for 30 min at 37°C followed by washings with PBS. Cells were re-fed every other day and counted after 7 days. PDs/day were calculated as log_2_ (# cells at the time of sub-culture/# cells plated)/days in culture. Live images of the cells were taken with a Leica DMIL LED inverted microscope (Leica Microsystems, Wetzlar, Germany). Keratinocyte hypermotility induced by TGFβ1-and ECM coating was assessed by IF using an anti-Laminin α3 antibody (Supplementary Table S3).

### 2.10 Soft agar assay

In a 9.5 cm^2^ cell culture dish, an acellular layer of 0.6% agar (Alfa Aesar, Thermo Fisher Scientific) in KSFM was overlaid with a top layer consisting of 7,500 cells resuspended in 0.3% agar in KSFM. Plates were stored at RT until the agarose solidified before being incubated at 37 °C for 18 days with regular re-feedings. Cell colonies were stained with 0.1% crystal violet.

### 2.11 Karyotype analysis

To assess chromosome abnormalities, exponentially growing, colcemid-treated (Thermo Fisher Scientific), and hypotonic (KCl) keratinocytes were fixed in freshly prepared, ice-cold methanol:acetic acid (3:1) fixative before Giemsa staining. Thereafter, metaphases were analyzed under the light microscope with the Genikon software (Nikon, Tokyo, Japan). At least five metaphases were analyzed per cell culture.

### 2.12 *In vitro* differentiation

Keratinocytes grown for 2 days in basal KSFM (0.1 mM Ca^2+^) were seeded at a density of 8 × 10^4^ cells/21.5 cm^2^ in basal KSFM. 24 h later, cells were subjected to a Ca^2+^ switch (1.8 mM) to induce differentiation for an additional 3 days. Cells were then imaged, extracted for RNA, and fixed in 4% buffered formaldehyde for further analysis.

### 2.13 3D-cultures

Keratinocytes were seeded in antibiotic-free basal KSFM on 12 mm polycarbonate inserts with 0.4 µm pores (Nunc, Thermo Fisher Scientific) in a volume of 310 μL at 1.55 × 10^5^ cells per insert. These inserts were set at the lowest position in 24-well carrier plates (Nunc) with 900 µL of antibiotic-free basal KSFM outside the insert. After 3 days, medium was switched for 18 h to 3D-Prime-Airlift medium (CnT-PR-3D, CELLnTEC, Bern, Switzerland). Subsequently, the inserts were lifted to the highest position in the carrier plate, the medium inside the insert was removed, and the outside of the insert was replenished with 1.6 mL of CnT-PR-3D to establish an air-liquid interface culture. CnT-PR-3D was renewed every other day for a total incubation time of 10 days.

### 2.14 Histology and immunohistochemistry (IHC)

Lip tissue and 3D cultures were fixed at RT in 4% buffered formaldehyde for 24 h and 2 h, respectively, prior to being subjected to dehydration using increasing ethanol concentrations. Thereafter, samples were passed through three changes in xylene and three changes in paraffin at 65°C before paraffin embedding. Two point 5 µm sections were obtained using a Reichert-Jung microtome (Leica Microsystems, Wetzlar, Germany) and were either stained with hematoxylin-eosin (H&E), Periodic Acid-Schiff (PAS) or processed for IHC.

For IHC, sections were deparaffinized in xylene and rehydrated through descending concentrations of ethanol and ddH_2_O. Tris-EDTA buffer, pH 9.0, was used for antigen retrieval by incubating the sections in a microwave oven at near-boiling temperature for 15 min. After cooling and further rinsing with TBS, 2.5% goat serum (Vector Laboratories) was applied for 30 min at RT to block non-specific binding. Primary antibodies (Supplementary Table S3) diluted in blocking solution were incubated for 1 h at RT. After three washes with TBS, polymer-based HRP-conjugated secondary antibodies (ImmPRESS^®^ goat anti-mouse or anti-rabbit IgG-Polymer Detection Kit, Peroxidase, Vector Laboratories) were added, followed by a further wash with TBS. Signal visualization was performed using the ImmPACT^®^ DAB Peroxidase Substrate (Vector Laboratories). Cell nuclei were counterstained with hematoxylin, and slides were mounted with Aquatex (Sigma-Aldrich).

### 2.15 Infection of 3D skin models with C. albicans

10 mL cultures of *Candida albicans* in broth (BHI) (Difco™, Becton Dickinson, Franklin Lakes, NJ, United States) plus 0.5% (w/v) glucose were incubated overnight at 37°C. Candidal cells were harvested by centrifugation at 3,000 rpm for 5 min and the pellet was washed 3× with 10 mL sterile PBS and finally resuspended in PBS to give 4 × 10^7^ colony forming units per ml (cfu/mL). 3D cultures of 19K-Ep/T were inoculated with 2 × 10^6^ cfu of a candidal strain suspension on day 9 of the 3D culture and incubated for another 24 h at 37°C. A non-infected PBS control was also included for comparison.

### 2.16 Scratch wound assay

19K-Ep/T were seeded at a density of 4 × 10^4^ cells per well in a 96-well ImageLock™ Microplate (Sartorius, Göttingen, Germany) in basal KSFM. 24 h post seeding, cell monolayers were pre-treated for 2 hours with 0.1 ng/mL of EGF or transforming growth factor α (TGFα) (both from Thermo Fisher Scientific) before being scratched with a 96-pin Incucyte WoundMaker (Sartorius). Images of the scratches were captured every hour using the IncuCyte S3 Live-Cell Analysis System (Sartorius). Scratch assays were analyzed with ImageJ (Fiji), employing the formula: Wound Closure = (area of initial wound-area of closing wound)/area of initial wound.

### 2.17 Statistics

Experiments were performed at least three times in multiple replicates. Data were analyzed using Prism 10 (GraphPad, La Jolla, CA, United States) and are reported as means ± standard deviation. Multiple comparisons were performed using one- or two-way analysis of variance with Tukey’s *post hoc* test. Data were considered significant when *p* < 0.05.

## 3 Results

### 3.1 Immortalization of lip-derived keratinocytes

**FIGURE 1:**
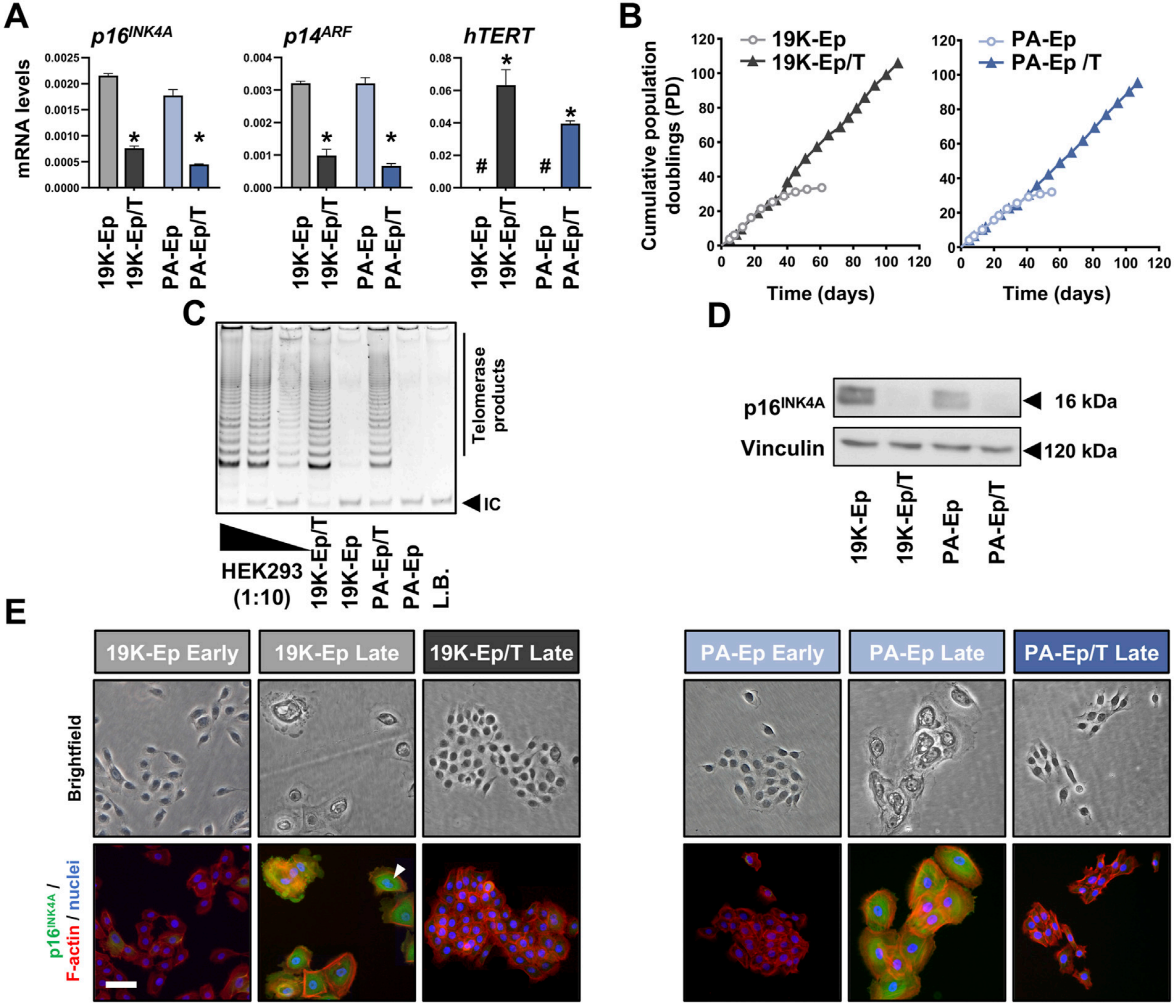
Immortalization of lip keratinoctyes. **(A)** qPCR analyses of *p16* ^*INK4A*^, *p14* ^*ARF*^, and *hTERT* in the keratinocytes 19K-Ep, 19K-Ep/T, PA-Ep, and PA-Ep/T. #: not detectable; *: *p* < 0.05. **(B)** Extended lifespan in 19K-Ep/T and PA-Ep/T compared to the primary cells, which senesced after 35 PDs. **(C)** TRAP assay shows increased telomerase activity in 19K/T-Ep and PA/T-Ep compared to their parental cell cultures. 2,500 (Lane 1), 250 (Lane 2), and 25 (Lane 3) HEK293 cells were used as positive controls. L.B. Lysis Buffer; IC: internal standard control band. **(D)** Immunoblot confirms p16^INK4A^ knockdown in late-passage 19K-Ep/T and PA-Ep/T compared to the late-passage primary cells. kDa: kilo Dalton. **(E)** Brightfield (top) and IF (bottom) for p16^INK4A^ (green), actin (red), and nuclei (blue) for early- and late-passage primary vs. late immortalized cells. “Early” indicates cells at low PDs, and “late” indicates cells at high PDs. Arrowhead shows a multinucleated cell. Scale bar: 50 µm. Note that only late 19K-Ep/T and PA-Ep/T were used because retention of p16^INK4A^ knockdown was assessed.

We sought to immortalize primary lip keratinocytes derived from a healthy (19K-Ep) and a non-syndromic CLP donor (PA-Ep) by employing a retroviral vector engineered to simultaneously express an shRNA targeting the *p16* ^*INK4A*^ and *p14* ^*ARF*^ mRNA (both encoded by the *CDKN2A* gene), as well as the cDNA of *hTERT* (Smith et al., 2016). To assess whether the resulting stable and polyclonal cell lines 19K-Ep/T and PA-Ep/T were indeed immortal, we initially analyzed *p16* ^*INK4A*^, *p14* ^*ARF*^, and *hTERT* transcript levels (Figure 1A). Compared to the respective parental keratinocytes, 19K-Ep/T and PA-Ep/T displayed significant downregulation of *p16* ^*INK4A*^ and *p14* ^*ARF*^, while *hTERT* levels were strongly increased. Since an extended lifespan is the most relevant aspect of immortalization, we evaluated the replicative lifespans of 19K-Ep/T and PA-Ep/T and compared them to the ones of their primary counterparts (Figure 1B). While the primary cells only proliferated for about 30 PDs, the immortalized strains easily reached up to 100 PDs with constant growth rates. We further measured increased telomerase activities in immortalized vs. parental keratinocytes (Figure 1C). Additionally, only late-passage 19K-Ep and PA-Ep displayed robust p16^INK4A^ protein expression (Figure 1D, E; Supplementary Figure S1A, B), which was found associated with cells displaying signs of senescence, including a flattened, enlarged, and multinucleated morphology (Figure 1E). Collectively, our first set of data demonstrated that lip-derived keratinocytes could be efficiently immortalized.

### 3.2 Immortalized lip keratinocytes preserve genomic stability

**FIGURE 2:**
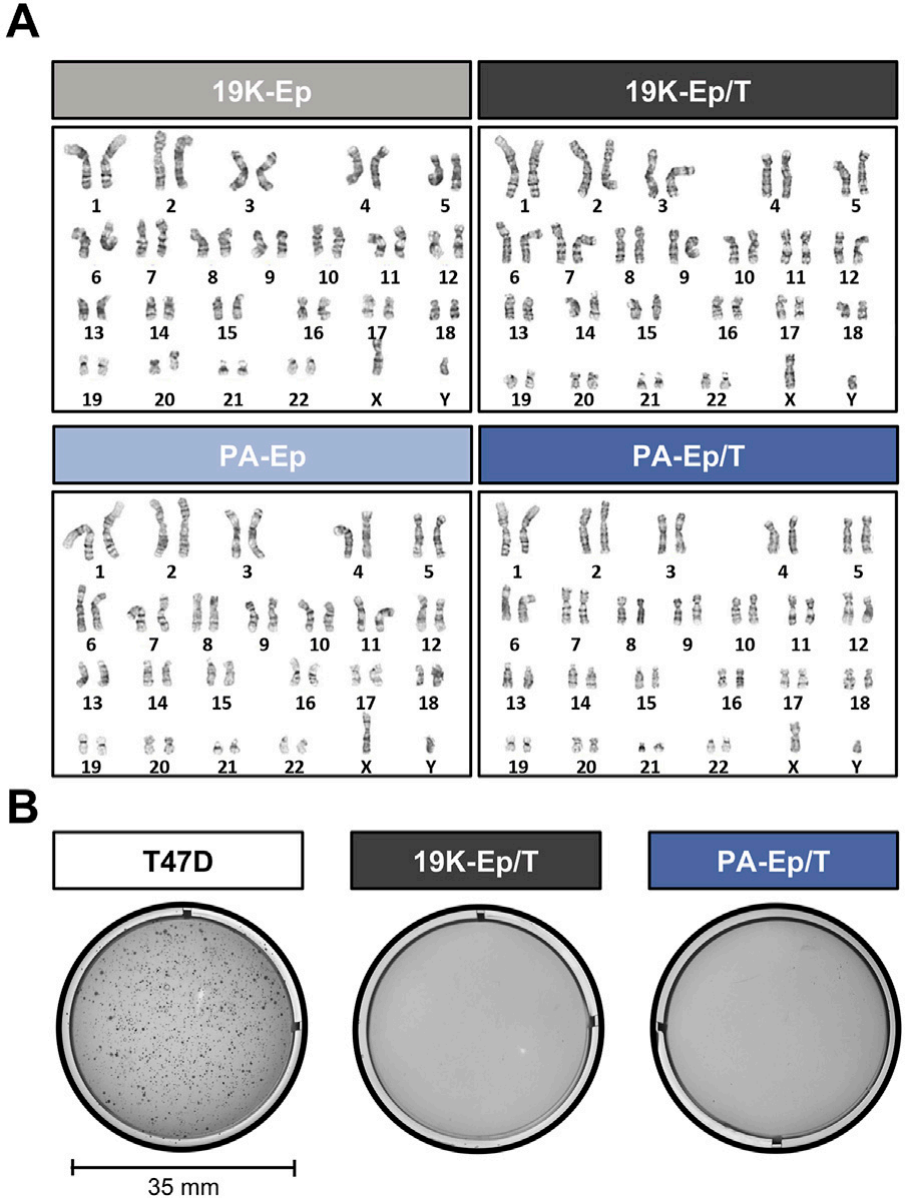
Genetic stability. **(A)** Comparative karyotype analysis of primary (left) and immortalized (right) late-passage keratinocytes reveals normal chromosomal number and structure. “Late” indicates cells at high PDs. **(B)** Soft agar assay demonstrates the non-transformed state of late-passage 19K-Ep/T and PA-Ep/T cell lines. Note that the breast cancer cell line T47D formed colonies when cultured in soft agar. “Late” indicates cells at high PDs.

To exclude that the virus-mediated immortalization approach caused any chromosomal modifications, we performed cytogenetic analyses. Representative karyograms of late-passage immortalized keratinocytes and their corresponding early-passage primary cells are shown in Figure 2A. 19K-Ep/T and PA-Ep/T maintained a normal human diploid karyotype 46,XY without any discernible chromosomal aberration compared to 19K-Ep and PA-Ep, respectively. To further prove the absence of any tumorigenic potential gained during immortalization and serial passaging, we conducted a soft agar assay. Late-passage 19K-Ep/T and PA-Ep/T remained anchorage-dependent as they failed to grow in soft agar, which contrasted with the breast cancer cell line T47D (anchorage-independent growth) (Figure 2B). These results suggested that the immortalized keratinocyte lines appear chromosomally stable and not malignantly transformed.

### 3.3 Immortalized lip keratinocytes maintain their normal morphology and properties

**FIGURE 3:**
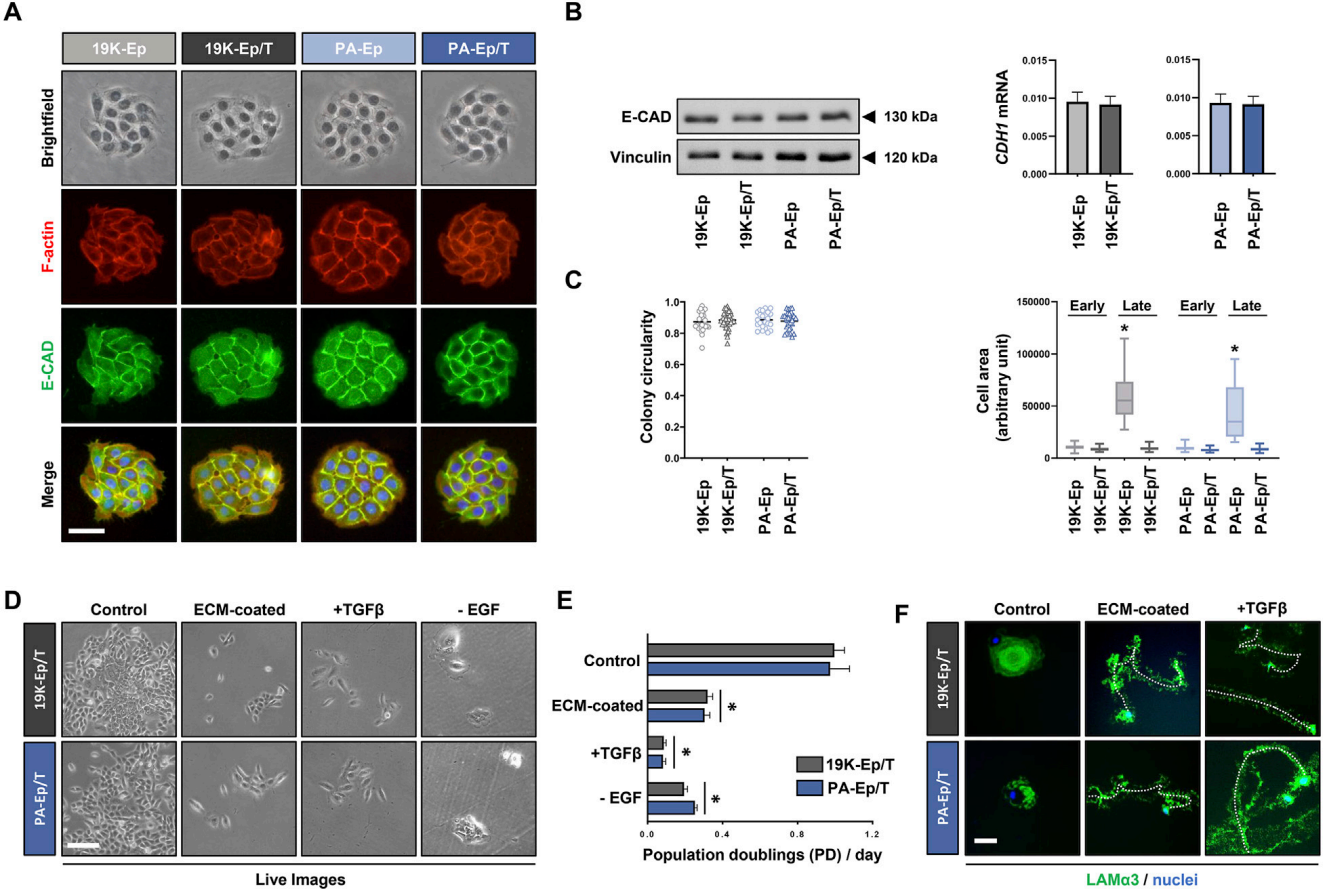
Maintenance of typical keratinocyte properties. **(A)** Brightfield as well as IF staining for E-Cadherin (E-CAD, green), actin (red), and nuclei (blue). Scale bar: 50 µm. **(B)** Immunoblot for E-CAD (left) and qPCR analysis of *CDH1* transcript levels (right) in the immortalized and primary cells. **(C)** Quantification of the colony circularity (left) and single-cell areas (right) in early- and late-passage primary and immortalized keratinocytes. Note that the late-passage primary keratinocytes increased their single-cell area indicative of senescence. *: *p* < 0.05. Note that “early” indicates cells at low PDs, and “late” indicates cells at high PDs. **(D)** Live images of 19K-Ep/T and PA-Ep/T cultured in regular KSFM or cultured on dishes pre-coated with fibroblast conditioned medium (ECM-coated), in the presence of TGFβ1 or the absence of EGF. Note that compared to control, all three conditions impaired keratinocyte growth. Scale bar: 100 µm. **(E)** Quantification of the growth assays by cell counting 7 days after seeding the cells on the described conditions revealed a significant decrease in the PDs/day on the ECM pre-coated dishes, in the presence of TGFβ1, or the absence of EGF. *: *p* < 0.05. **(F)** 19K-Ep/T and PA-Ep/T hypermotility induced by ECM pre-coating and TGFβ1 as assessed by IF for Laminin α3 (LAMα3)-positive migration tracks (green) compared to control. DAPI was used to counterstain cell nuclei (blue). A dashed line follows a cell along the migration track. Scale bar: 50 µm.

Next, we were keen to assess whether 19K-Ep/T and PA-Ep/T retained typical keratinocyte morphology. We stained cell colonies formed by the immortalized and parental keratinocytes for E-Cadherin (E-CAD) and F-actin, which revealed indistinguishable colony shapes among the four strains with prominent E-CAD expression at adherens junctions between cells (Figure 3A). Furthermore, protein and mRNA levels of E-CAD in 19K-Ep/T and PA-Ep/T were indistinguishable from those of their parental primary cells (Figure 3B). Complementary gene expression analysis of other intercellular junction components also showed similar expression levels of *Occludin* (tight junctions) and *Desmocollin-1* (desmosomes) in 19K-Ep/T and PA-Ep/T compared to primary cells (Supplementary Figure S2). A quantitative determination of the colony circularities (cc) and the single cell areas of early-passage keratinocytes corroborated our results that 19K-Ep/T and PA-Ep/T are identical to their primary counterparts regarding cc and cell areas. Only the late-passage primary keratinocytes displayed significantly increased cell areas compared to both early- and late-passage 19K-Ep/T and PA-Ep/T (Figure 3C), which was in agreement with our previous observations (Figure 1E).

19K-Ep/T and PA-Ep/T can only serve as clinically relevant cell models as long as they behave identically to their parental primary keratinocytes. Primary keratinocytes depend on EGF for their *in vitro* proliferation (Rheinwald and Green, 1977) and undergo a growth arrest/hypermotility response upon engaging specific extracellular matrices (ECM) as substrates and treatment with TGFβ1 (Natarajan et al., 2006). We wanted to test the retention of these typical keratinocyte traits (Supplementary Figure S3) in 19K-Ep/T and PA-Ep/T. Pre-coating dishes with fibroblast conditioned medium (ECM-coated), stimulation with TGFβ1, as well as lack of EGF in the growth medium, elicited stark growth impairments of 19K-Ep/T and PA-Ep/T when compared to control conditions. This was assessed by live imaging (Figure 3D) and corresponding cell counts after 7 days in culture (Figure 3E). Additional dishes with 19K-Ep/T and PA-Ep/T keratinocytes cultured under control, ECM-coated and TGFβ1-treated conditions were stained for Laminin α3 (Figure 3F). Laminin α3-positive migration tracks were detected in the immortalized keratinocytes only in response to the ECM substrates and TGFβ1 treatments. Therefore, we concluded that typical keratinocyte traits are maintained in 19K-Ep/T and PA-Ep/T.

### 3.4 Immortalized lip keratinocytes display their expected differentiation potential

**FIGURE 4:**
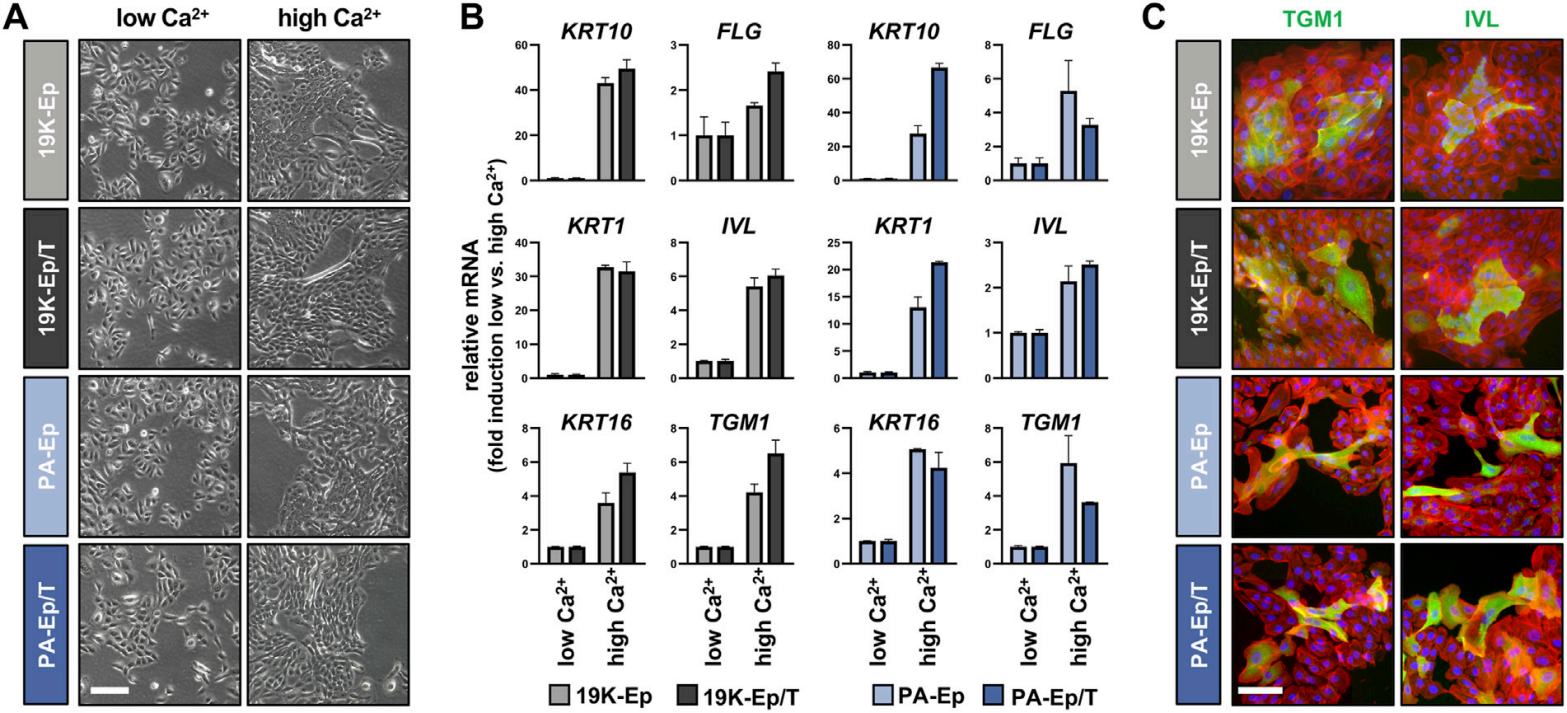
Regular differentiation potential in 2D. **(A)** Live images of primary and immortalized keratinocytes 3 days after induction of differentiation by high Ca^2+^ (1.8 mM, right column) compared to low Ca^2+^ (0.1 mM, left column). Scale bar: 100 µm. **(B)** qPCR analysis of typical differentiation markers in 19K-Ep vs. 19K-Ep/T and PA-Ep vs. PA-Ep/T reported as fold induction (ΔΔCt) of normalized levels in high vs. low Ca^2+^ (set to 1) conditions. Note that all markers showed a substantial upregulation upon Ca^2+^-switch. **(C)** IF pictures of Ca^2+^-differentiated 19K-Ep, 19K-Ep/T, PA-Ep, and PA-Ep/T cultures for the general differentiation markers Transglutaminase 1 (TGM1, green) and Involucrin (IVL, green) as well as actin (red) and nuclei (blue). Scale bar: 50 µm.

One of the hallmarks of keratinocytes is their ability to undergo an epithelial differentiation program. Therefore, we compared the differentiation potential of 19K-Ep/T and PA-Ep/T to that of their parental counterparts. All keratinocyte cultures shifted from loosely packed colonies in low Ca^2+^ to tightly packed ones in high Ca^2+^ concentrations (Figure 4A). Accompanying these morphological changes, the Ca^2+^-switch triggered a robust induction of typical differentiation markers, including Keratin 1 (*KRT1*), *KRT10*, *KRT16*, Filaggrin (*FLG*), Involucrin (*IVL*), and Transglutaminase 1 (*TGM1*), without any substantial deviations between the immortalized and the parental lines (Figure 4B; Supplementary Figure S4A). The keratinocyte cultures were also stained for TGM1 and IVL, and protein levels were quantified by western blot, which confirmed the similar differentiation potential of immortalized and parental keratinocytes in response to increased Ca^2+^ (Figure 4C; Supplementary Figure S4B).

**FIGURE 5:**
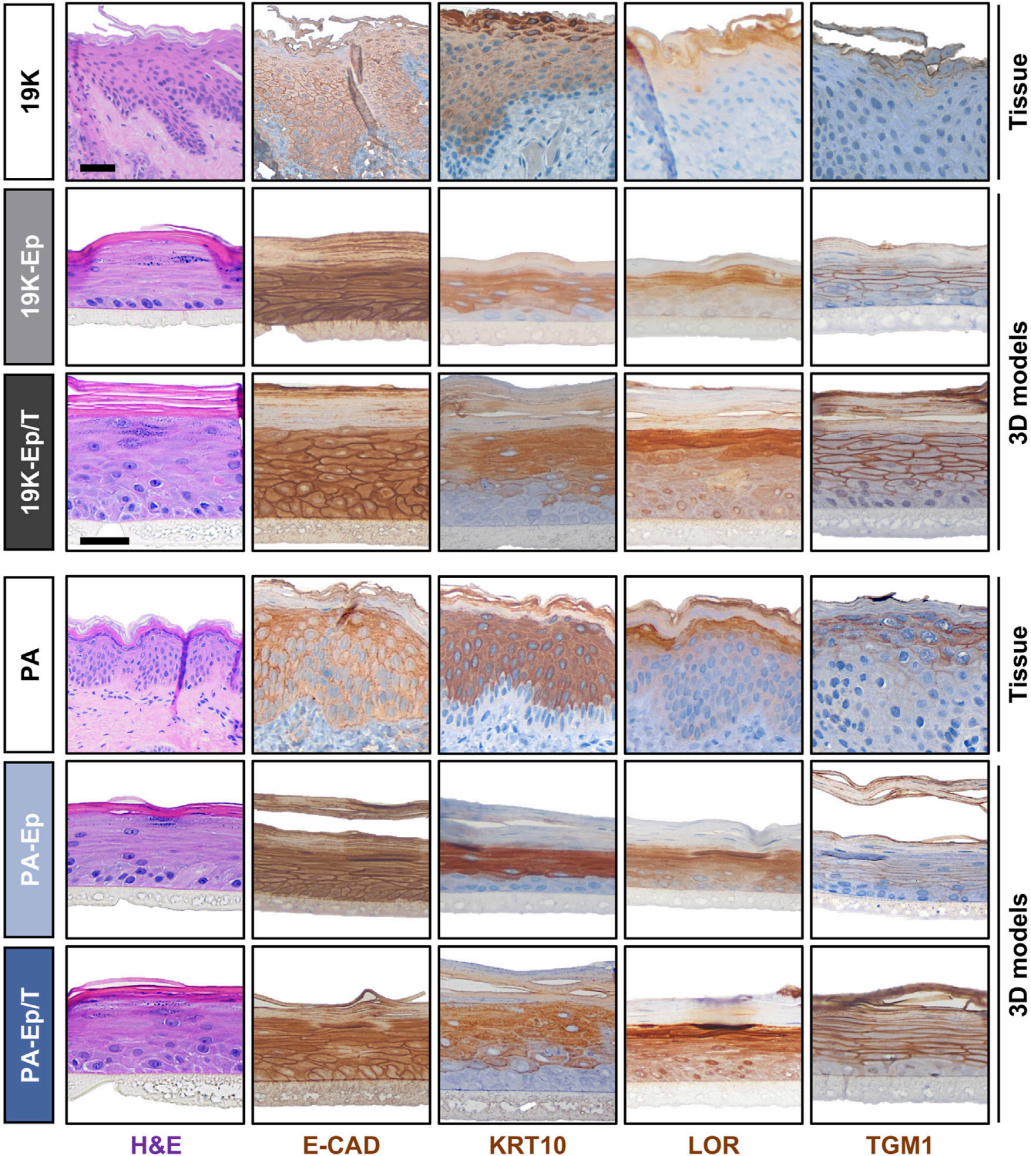
Establishment of 3D lip models. H&E and IHC staining of primary lip tissue (top line) and 3D lip models derived from primary (middle line) and immortalized (bottom line) keratinocytes for the patients 19K (top panel) and PA (bottom panel). Note identical morphology (H&E) and expression pattern of the various markers E-Cadherin (E-CAD), Keratin 10 (KRT10), Loricrin (LOR), and Transglutaminase 1 (TGM1) between primary keratinocytes, immortalized keratinocytes, and native lip tissues. Also note that the 19K healthy lip tissue displays damaged outer epithelial layers, which might be caused by the trauma. However, strong Keratin10-positivity still suggests a skin-like identity of 19K. Scale bar (tissue): 100 μm; scale bar (3D structure): 50 µm.

Prompted by these observations, we used 19K-Ep/T, PA-Ep/T, and their respective primary counterparts to establish three-dimensional (3D) lip models, allowing a comparison to the original lip tissue biopsy (Figure 5). H&E staining revealed that 19K-Ep/T and PA-Ep/T formed 3D cultures identical to their primary counterparts, all forming stratified squamous keratinized epithelia, which closely resembled their corresponding tissues. IHC analyses further illustrated that the expression pattern of E-CAD as well as of the differentiation markers KRT10, Loricrin (LOR), and TGM1 in 19K-Ep/T and PA-Ep/T faithfully recapitulated their localization in primary cell-derived 3D models as well as in the parental lip tissues. Collectively, all our results validated the newly established immortalized cell lines as reliable and clinically relevant lip (healthy and CLP) keratinocytes that profoundly retain original lip tissue characteristics and traits.

### 3.5 Immortalized lip keratinocytes as *in vitro* tools to study lip abnormalities

**FIGURE 6:**
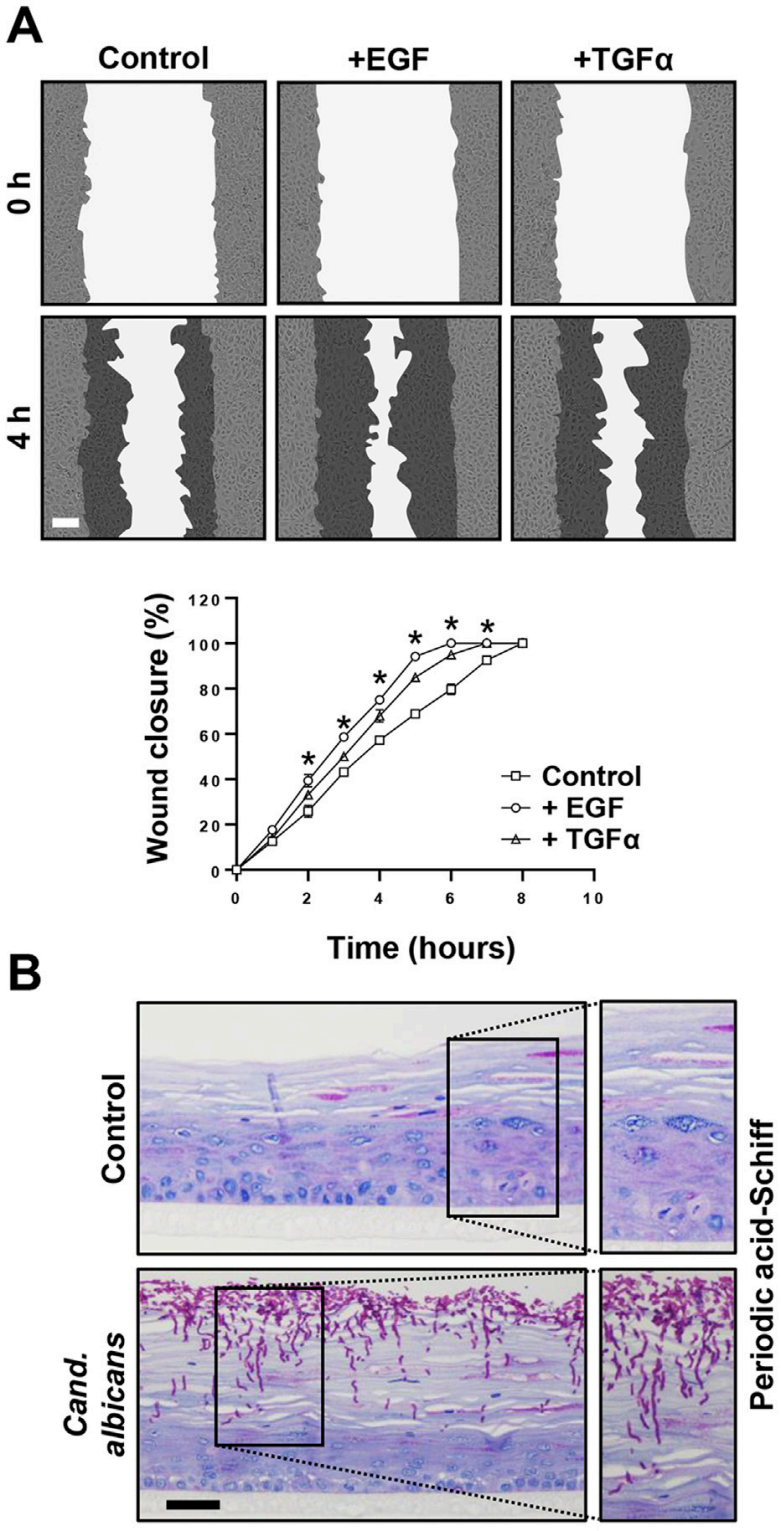
Promising screening platforms. **(A)** Live imaging pictures and quantification of scratch assays of 19K-Ep/T in the presence of 0.1 ng/mL EGF and 0.1 ng/mL TGFα at indicated time points. Note that the presence of EGF and TGFα stimulated the closing of the cell-free gap. *: *p* < 0.05. Scale bar: 50 µm. **(B)** 19K-Ep/T 3D lip models inoculated with *Candida albicans* for 24 h and visualized by PAS staining revealing pathologic hyphae penetrating the epithelium. Scale bar: 50 µm.

The last set of experiments was devoted to potential future applications of the immortalized lip keratinocytes. Initially, we used 19K-Ep/T in scratch assays to study wound healing, which is of high clinical relevance both for trauma- and surgery-related lip wounds, such as lacerations and corrective cleft surgeries, respectively. We selected a panel of growth factors and tested their abilities to modulate the migratory behavior of 19K-Ep/T keratinocytes. As can be appreciated in Figure 6A, the untreated control cells exhibited complete wound closure after 8 h, while the TGFα- and EGF-treated groups demonstrated a significantly sped-up migratory behavior resulting in faster wound closures. These results underscored the accelerated migratory behavior of lip keratinocytes upon TGFα and EGF treatments, which aligned with previous reports using non-lip-derived keratinocytes (Seeger and Paller, 2015).

Finally, we used the 3D lip cultures as *in vitro* models to establish them as robust systems for investigating microbial infections. This approach holds clinical significance given that the lips are highly vulnerable to infections, which often results in a temporarily disfiguring appearance. We initially used *C. albicans* and infected 3D lip cultures (Figure 6B). PAS staining revealed the prominent presence of pathological, hyphae-forming *Candida*, which actively invaded and penetrated the upper layers of the stratified squamous and keratinized lip epithelium. This model system should allow future studies to better understand the molecular mechanisms of infection and the cellular responses of the epithelium upon encountering the pathogens, enabling the potential discovery of novel treatment strategies.

Taken together, our data convincingly substantiated the immense potential immortalized lip keratinocytes have as optimal screening platforms for multiple readouts that might translate into noticeable benefits for individuals suffering from lip abnormalities, such as CLP, traumas, infections, and lip cancers.

## 4 Discussion

Primary cells are widely recognized as the optimal choice for *in vitro* models. However, they have some drawbacks that hinder their large-scale implementation in translational research. The use of primary cells is often associated with ethical issues, technical challenges, high costs, and limited availability. Immortalized cells can overcome some of these hurdles, yet the accessibility of immortalized keratinocytes is restricted and only encompasses certain tissues, including the foreskin, floor of the mouth, gingiva, and lung (Dickson et al., 2000; Degen et al., 2012; Moffatt-Jauregui et al., 2013; Smith et al., 2016; Smits et al., 2017). Here, we present data on the establishment, thorough characterization, and potential application of novel healthy and non-syndromic CLP lip-derived immortalized keratinocyte lines. To the best of our knowledge, this marks the first instance of the introduction of such cells into the scientific community, which represents an important step toward a better understanding of lip-related diseases and malformations. Various research fields, including oral pathology and dentistry, are in line to benefit from the availability of immortalized lip keratinocytes. 3D cultures of such cells might represent the preferred choice for translational research endeavors, such as grasping the molecular basis of mucositis, a common complication of head and neck cancer chemotherapy and radiation, assessing the toxicity of novel toothpastes and mouthwashes, or evaluating cost-effectively, potential treatments for actinic cheilitis before processing to expensive pre-clinical studies.

To immortalize lip keratinocytes, a one-step approach was adopted, which relied on the introduction of hTERT and the simultaneous knockdown of the cell cycle inhibitor p16^INK4A^ (Smith et al., 2016). We chose this protocol to avoid the necessity of two transduction cycles as traditionally employed (Dickson et al., 2000). This approach should minimize the risk of cellular stress and genetic alterations of the target cells, which might be linked to aberrant phenotypes. Indeed, our findings strongly supported the preservation of the original lip characteristics in the immortalized cell lines (Figures 1–5), which underlined their potential as a robust and reliable tool for human *in vitro* lip modeling.

The lip is a prominent facial feature of significant medical and cosmetic importance. However, a challenge for lip-focused research is the mucocutaneous nature of its lining. Depending on the tissue biopsy, which cannot be standardized, explant outgrowths can result in labial skin, mucosa, or mixed keratinocyte cultures (Degen et al., 2018; Rihs et al., 2024). Being aware of this phenomenon, we could not unambiguously authenticate the initial 19K-Ep and PA-Ep cells as pure skin or mucosa cells. However, our histological analyses pointed towards a more skin-like phenotype of both 19K and PA since the corresponding 3D models, as well as the original lip tissues, displayed prominent features of keratinized epithelia (Figure 5). It should be mentioned that if needed, immortalized lip skin and/or mucosa keratinocytes could be obtained either by single-cell clonal analysis of a mixed cell population with subsequent analysis of skin (*e.g*., *KRT10*) and mucosa (*e.g*., *KRT13*) markers or by a precise tissue separation during cheiloplasty (Rihs et al., 2024). Pure lip skin and mucosa keratinocytes would represent the optimal source for the establishment of human lip vermilion *in vitro* models. So far, the limited accessibility of lip cells in the scientific community forced the use of alternative keratinocytes derived from other tissues (*e.g*., skin keratinocytes from breast reductions and mucosa keratinocytes from undefined oral mucosa) instead of lip-derived keratinocytes for modeling the human vermilion (Peramo et al., 2012; Bayar et al., 2016; Kobayashi et al., 2023). While acknowledging these efforts, it should be noted that the lip skin has distinctive features, including a thinner cornification (Tamura et al., 2016), a higher trans-epidermal water loss (Tagami, 2008) and most likely a unique lipid composition (Merleev et al., 2022) compared to other areas of body skin, highlighting the unique characteristics of lip keratinocytes.

As mentioned above, future research focused on lip defects/malformations or more generally on the oral cavity (*e.g.,* lip mucosa), might highly benefit from the availability of clinically relevant immortalized lip keratinocytes. Herein, we provided proof-of-concept data on their successful use as platforms for wound healing investigations in 2D (Figure 6A) or for microbial infections studies in 3D (Figure 6B). Besides the general population, wound healing is highly relevant for CLP-affected patients as a fraction of them develop excessive scar tissue after corrective lip surgery (van Beurden et al., 2005). Whether clefting and wound repair are conserved events that share common pathways and gene regulatory networks, as proposed (Biggs et al., 2015), remains to be fully elucidated. We are convinced that *in vitro* screening efforts to prospectively identify those CLP patients within a given patient cohort, who will develop wound healing complications and to match this phenotype to specific genes and factors is more feasible in immortalized than primary keratinocytes. Another potential application for the immortalized lip keratinocytes is found in studies focusing on the interaction of the lip epithelia with specific microbes. As the lips are vulnerable to various infections and since CLP patients seem to experience an increased risk of them (Khan et al., 2023), such studies are highly welcomed as well. Our proof-of-concept study used the common, human commensal organism *C. albicans*, which is an opportunistic pathogen that can cause severe damage in immunocompromised hosts (Tong and Tang, 2017), as well as in patients with anatomical alterations in the oral cavity, such as cleft-affected individuals (Khan et al., 2023). 3D models using immortalized lip keratinocytes provide the ideal platform to (i) study dysbiosis; (ii) screen for antimicrobial agents; or (iii) assess the patient cell-specific immune response (Figure 6B). 3D models are preferred over conventional 2D cultures as they more faithfully recapitulate the *in vivo* situation. However, they require large quantities of cells. Therefore, immortalized keratinocytes are better suited for these experiments than primary cells. Finally, immortalized healthy and CLP lip keratinocytes are an asset for elucidating specific gene functions by genome editing using CRISPR/Cas9. This technology involves multiple passaging of cells because clonal selection might be required, which renders primary keratinocytes with a finite lifespan not highly suitable for this approach. Although some publications are applying CRISPR/Cas9 in primary keratinocytes (Fenini et al., 2018; Mercado et al., 2019; Galvez et al., 2020; Grossi et al., 2020), such studies remain challenging and scarce. Our laboratory has recently used the immortalized foreskin-derived line N/TERT-1 and the oral mucosal line OKF6/TERT-2 for studying the function of the CLP candidate gene Interferon Regulatory Factor 6 (*IRF6*) (Girousi et al., 2021). While the absence of IRF6 in N/TERT-1 and OKF6/TERT-2 confirmed the phenotypes observed in Irf6-deficient mice (Ingraham et al., 2006), we have to acknowledge the limitation of using non-lip-derived cells for our study. The use of immortalized lip keratinocytes would have been more clinically relevant and appropriate.

As the process of craniofacial development is highly conserved across vertebrate model organisms, most of the molecular knowledge about CLP is based on animal studies. However, there are certain differences between humans and animals, including specific craniofacial tissue morphologies and their interactions (Yu et al., 2017), variations in the transcriptome (Fougerousse et al., 2000) during development as well as manifestations of organism-specific cleft phenotypes (Gritli-Linde, 2008; Van Otterloo et al., 2016). Considering the urge to minimize animal testing following the 3R initiative (Replacement, Reduction, and Refinement), there is a clear need for alternative clinically relevant human *in vitro* tools. We feel confident that our immortalized keratinocytes can fulfill this demand. Their stability and dependability have the potential to encourage collaborative research to enhance our understanding of the complex field of healthy and diseased lip biology, covering topics from skincare and dentistry to craniofacial anomalies, such as CLP.

## Data Availability

The datasets generated and/or analyzed during the current study are available from the corresponding author on reasonable request.
