# Supplementary material for: Immortalization of patient-derived lip cells for establishing 3D lip models
Source: Front Cell Dev Biol. 2024 Nov 4;12:1449224. doi: 10.3389/fcell.2024.1449224 (PMC11570282; doi:10.3389/fcell.2024.1449224)
Supplement: Supplementary file 1 [file DataSheet1.docx]

Supplementary Material

**Immortalization of patient-derived lip cells for establishing 3D lip models**

Farah Mansour, Ludovica Parisi, Silvia Rihs, Isabelle Schnyder, Giorgio C La Scala, Nijas Aliu, Christos Katsaros, and Martin Degen*

*** Correspondence:** Martin Degen

martin.degen@unibe.ch

# Supplementary Figures and Tables

## Supplementary Figures

##
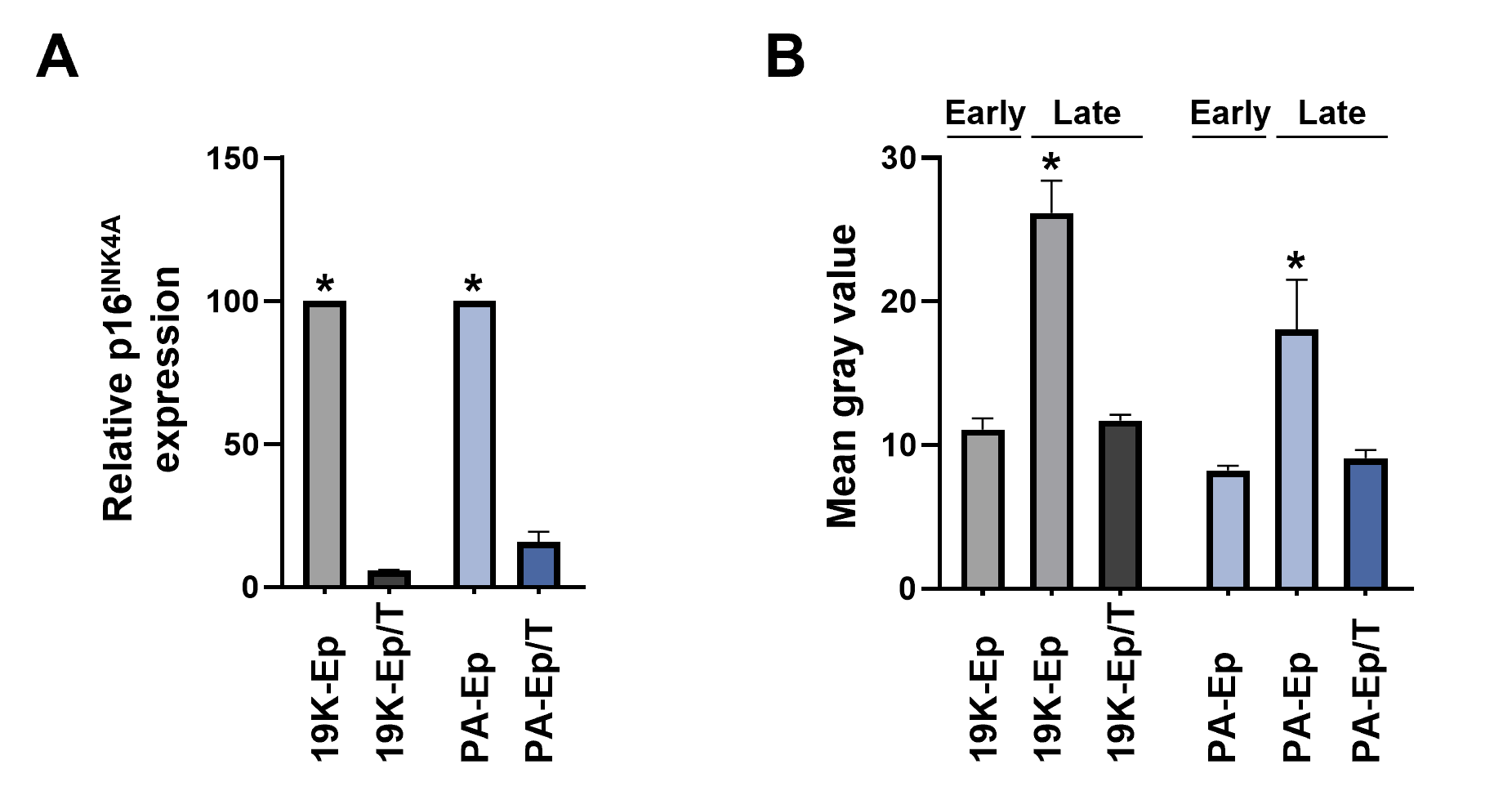


**Supplementary Figure 1. (A)** Western blot quantification of p16^INK4A^ levels in primary and immortalized cells normalized to Vinculin. *: p < 0.05. **(B)** Quantification of p16^INK4A^ fluorescence intensity in early- and late-passage primary and immortalized keratinocytes. *: p < 0.05. Note that “early” indicates cells at low PDs, and “late” indicates cells at high PDs.

**
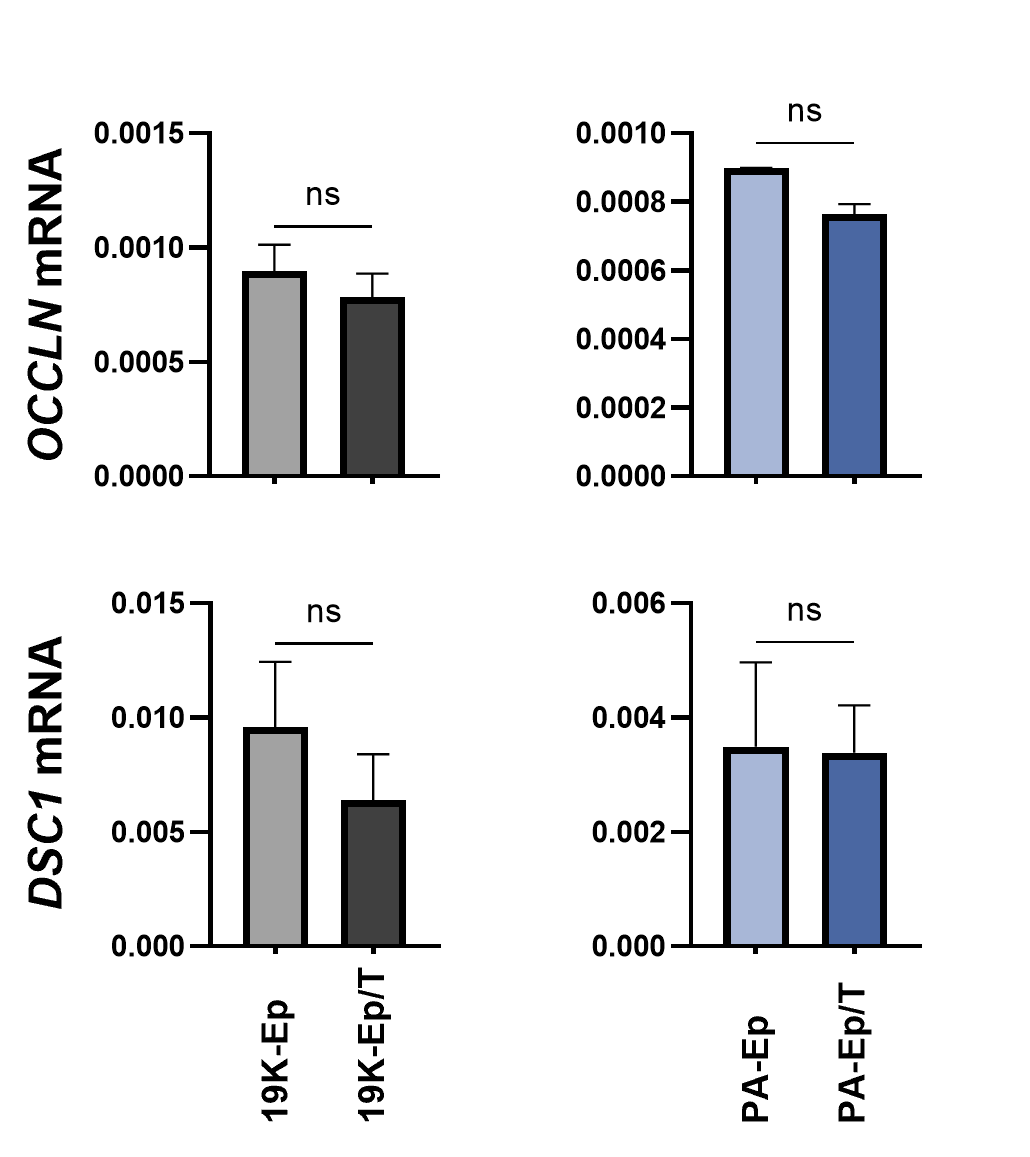
**

**Supplementary Figure 2.** qPCR analysis of *Occludin (OCCLN)* and *Desmocollin-1 (DSC1)* in 19K-Ep vs. 19K-Ep/T and PA-Ep vs. PA-Ep/T. Note that mRNA levels were similar between primary and immortalized cells. ns: not significant.

#
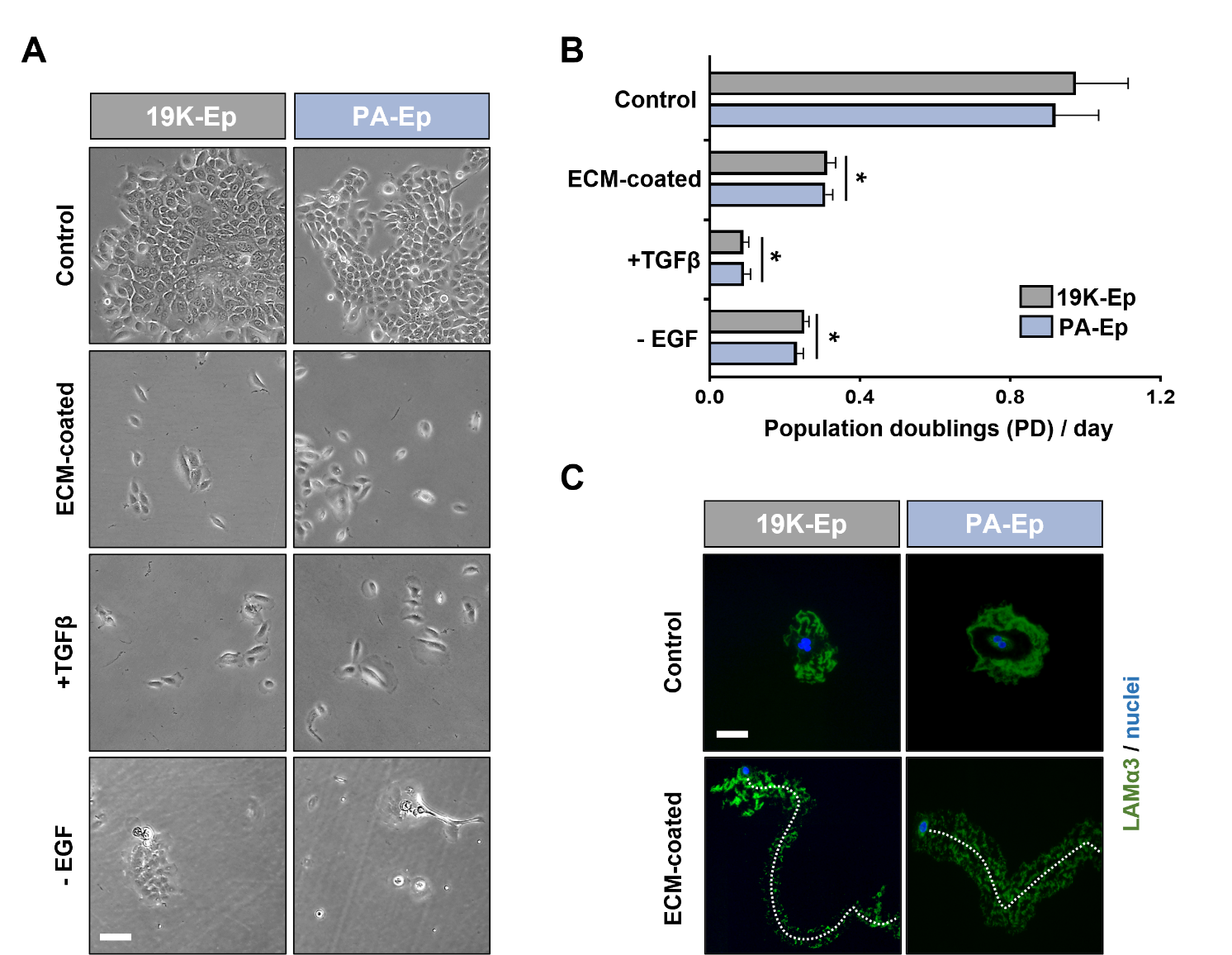


**Supplementary Figure 3. (A)** Live cell images showing low cell density, indicating a growth inhibition when primary cells are cultured on dishes pre-coated with fibroblasts conditioned medium, in the presence of +TGFβ or the absence of EGF. Scale bar: 50 μm. **(B)** Quantification of the growth assay of 19K-Ep and PA-Ep cultured in regular fully supplemented KSFM or cultured on dishes pre-coated with fibroblasts conditioned medium, in the presence of +TGFβ1 or the absence of EGF. Note that growth inhibition in the conditions tested is evidenced by a significant decrease in PD/day calculated after 7 days of culture, compared to control cultures. *: p < 0.05. **(C)** Laminin α3 immunostaining (green) 24 h after plating primary cells on control dishes (upper row) or dishes pre-coated with fibroblast conditioned medium (ECM-coated, lower row) showing induction of a hypermotility response. DAPI was used to counterstain cell nuclei (blue). Dashed lines: migration tracks. Scale bar: 50 μm.

**
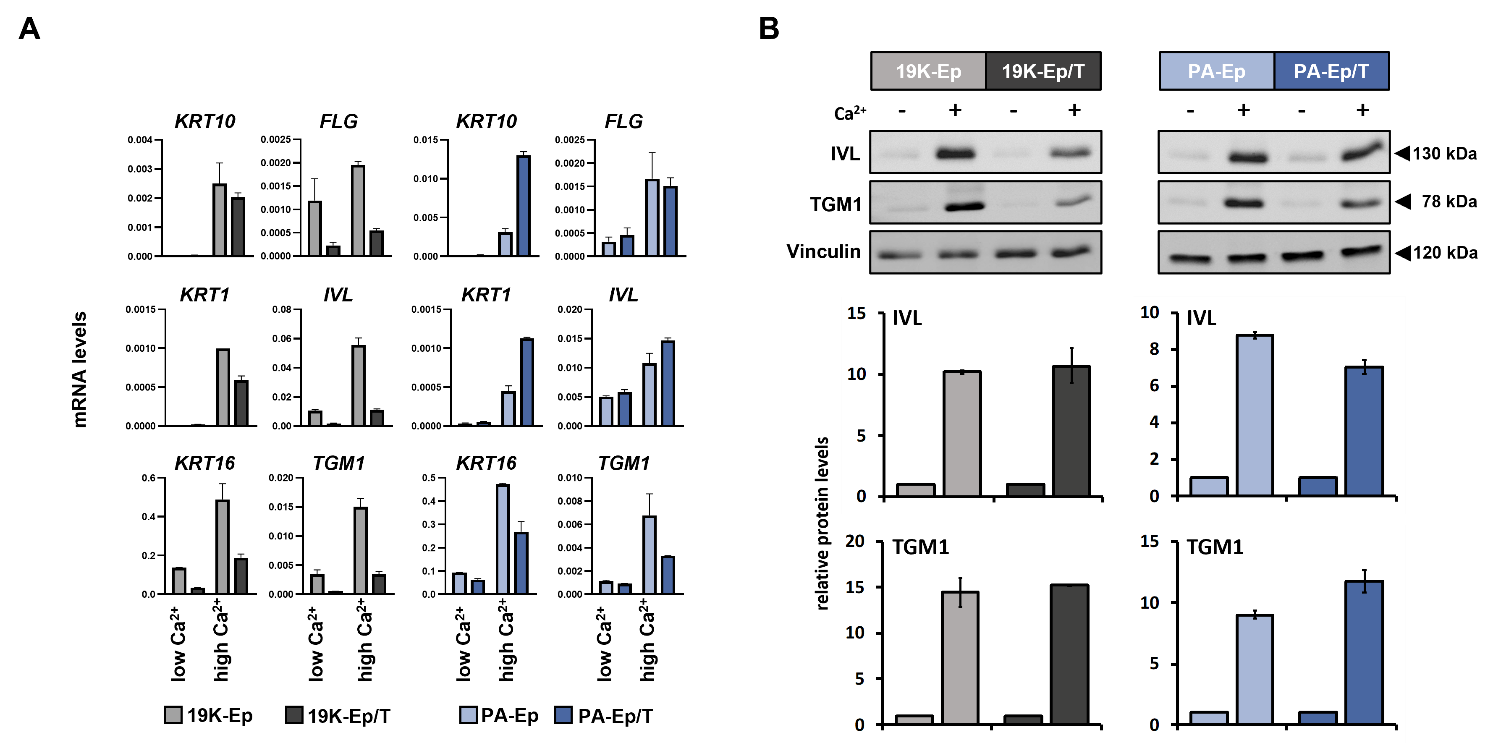
**

**Supplementary Figure 4.** **(A)**. qPCR analysis of typical differentiation markers in 19K-Ep vs. 19K-Ep/T and PA-Ep vs. PA-Ep/T reported as absolute values (ΔCt) in high vs. low Ca^2+^ conditions. (**B)** Immunoblots for IVL and TGM1 in the immortalized and parental cells in high vs. low Ca^2+^ conditions (top) as well as their quantification (bottom).

## Supplementary Tables

**Supplementary Table 1.** Characteristics of the cells used in the current study. 19K-Ep were obtained from a child sustaining a lip laceration (Degen et al., 2020), whereas PA-Ep keratinocytes were isolated from a non-syndromic (wild-type for *IRF6 (NM_006147.4), GRHL3 (NM_198173), and NME1_198175)* CLP patient (Degen et al., 2018;Degen et al., 2020). M, male; F, female; y, years; m, months; / not known.

**Supplementary Table 2.** Sequence of the qPCR primers used in this study. bp: base pairs.

**Supplementary Table 3.** List of antibodies used in this study and their sources.

# References

Degen, M., Girousi, E., Feldmann, J., Parisi, L., La Scala, G.C., Schnyder, I., Schaller, A., and Katsaros, C. (2020). A Novel Van der Woude Syndrome-Causing IRF6 Variant Is Subject to Incomplete Non-sense-Mediated mRNA Decay Affecting the Phenotype of Keratinocytes. *Front Cell Dev Biol* 8**,** 583115.

Degen, M., Wiederkehr, A., La Scala, G.C., Carmann, C., Schnyder, I., and Katsaros, C. (2018). Keratinocytes Isolated From Individual Cleft Lip/Palate Patients Display Variations in Their Differentiation Potential in vitro. *Front Physiol* 9**,** 1703.
